# Low-dose norepinephrine in combination with hypotensive resuscitation may prolong the golden window for uncontrolled hemorrhagic shock in rats

**DOI:** 10.3389/fphys.2022.1004714

**Published:** 2022-09-19

**Authors:** Yuanqun Zhou, Qinghui Li, Xinming Xiang, Yue Wu, Yu Zhu, Xiaoyong Peng, Liangming Liu, Tao Li

**Affiliations:** State Key Laboratory of Trauma, Burns and Combined Injury, Shock and Transfusion of Research Institute of Surgery, Daping Hospital, Army Medical University, Chongqing, China

**Keywords:** hypotension resuscitation, norepinephrine, uncontrolled hemorrhagic shock, organ function, golden treatment time

## Abstract

Hypotension resuscitation is an important principle for the treatment after trauma. Current hypotensive resuscitation strategies cannot obtain an ideal outcome for remote regions. With the uncontrolled hemorrhagic shock (UHS) model in rats, the effects of norepinephrine (NE) on the tolerance time of hypotensive resuscitation, blood loss, vital organ functions, and animal survival were observed. Before bleeding was controlled, only the LR infusion could effectively maintain the MAP to 50–60 mmHg for 1 h, while the MAP gradually decreased with prolonging time, even with increasing infusion volume. Low-dose NE during hypotensive resuscitation prolonged the hypotensive tolerance time to 2–3 h, and the effect of 0.3 μg/kg/min NE was the best. Further studies showed that 0.3 μg/kg/min NE during hypotensive resuscitation significantly lightened the damage of organ function induced by UHS via protecting mitochondrial function, while the LR infusion did not. At the same time, NE administration improved Hb content, DO_2_, and VO_2_, and restored liver and kidney blood flow. The survival results showed that low-dose NE administration increased the survival rate and prolonged the survival time. Together, low-dose NE during hypotensive resuscitation was suitable for the early treatment of UHS, which can strive for the golden window of emergency treatment for serious trauma patients by reducing blood loss and protecting vital organ functions.

## Introduction

Hemorrhagic shock is one of the leading causes of death following trauma (Kauvar et al., 2006). Fluid resuscitation is the first and key measure for the early treatment of traumatic hemorrhagic shock, which can restore the blood volume timely and effectively improve the success rate of treatment. In recent years, there have been several principles of early fluid resuscitation, including permissive hypotensive resuscitation, delayed resuscitation, and so on (Das et al., 2022). A large number of studies have shown that permissive hypotension resuscitation can reduce blood loss and then decrease the mortality rate, as compared with normotensive resuscitation in hemorrhagic shock patients (Woodward and Alsabri, 2021). However, the tolerance time is limited when using conventional resuscitation fluid such as lactated Ringer’s (LR) during permissive hypotension resuscitation. Our previous studies also demonstrated that 90 min was the maximal tolerance time for uncontrolled hemorrhagic shock (UHS) (Li et al., 2011). Unfortunately, the evacuation time is more than 90 min in remote regions or in disaster situations (Yang et al., 2015). Therefore, a proper strategy is urgently needed that can prolong the tolerance time for hypotensive resuscitation.

Norepinephrine (NE) is a vasopressor that can elevate arterial blood pressure effectively with the pharmacologic effects on α1 adrenoceptors. High-dose NE may result in excessive arteriolar vasoconstriction which subsequently leads to the disorder of microcirculation and tissue hypoxia. So, NE was recommended in the precondition of adequate volume resuscitation in the clinic with a dose of 0.05–0.3 μg/kg/min. In recent years, some studies have found that low-dose NE is beneficial to hemorrhagic shock during resuscitation in animals (Lee et al., 2009; Asfar and May 2022; Libert et al., 2022). However, the administration of vasopressors during the early phase of hemorrhagic shock remains controversial (Gupta et al., 2017; Hylands et al., 2017). The main consideration is that vasopressors carry a risk of excessive vasoconstriction which could aggravate tissue ischemia. Whether the low-dose NE is beneficial to UHS and prolongs the golden time of emergency treatment during permissive resuscitation is still unclear.

The purpose of the present study was to investigate the effects of NE (0.1, 0.3, 0.5 μg/kg/min) during hypotensive resuscitation on blood loss, hemodynamics, vital organ functions, and survival following UHS in rats. It aimed to search for a proper strategy for traumatic hemorrhagic shock during the prehospital stage.

## Materials and methods

### Ethical approval

All procedures were performed under the guidelines for the Care and Use of Laboratory Animals published by the US National Institutes of Health. This study was approved by the Research Council and Animal Care and Use Committee of the Research Institute of Surgery, Daping Hospital, Army Medical University (Chongqing, China). None of the authors are members of this committee.

### Animal preparation and uncontrolled hemorrhagic shock model establishing

Sprague-Dawley rats (220–260 g) were fed at the animal care center of Daping Hospital, Army Medical University (license No. SCXK [Yu]20170002). Rats were fasted for 12 h but allowed water *ad libitum* before the experiments. On the day of the experiment, rats were anesthetized with sodium pentobarbital (30 mg/kg). The right femoral arteries and veins were catheterized with a polyethylene catheter (outer diameter, 0.965 mm; inner diameter, 0.58 mm) for monitoring MAP and drug administration, respectively. Two catheters were inserted into the right carotid artery and jugular vein for hemodynamic measurement and infusion. To prevent clotting, the carotid artery and jugular vein were filled with normal (physiological) saline (0.9%) containing 30 U/ml of heparin. A model of UHS was induced by transection of the splenic parenchyma and one of the branches of the splenic artery and vein, as described previously by our research team (Li et al., 2012). Blood was allowed to flow freely into the abdominal cavity. When the MAP decreased to 40 mmHg (30–40 min), the UHS model was established for the subsequent experiments.

### Experimental phases and management

**FIGURE 1:**
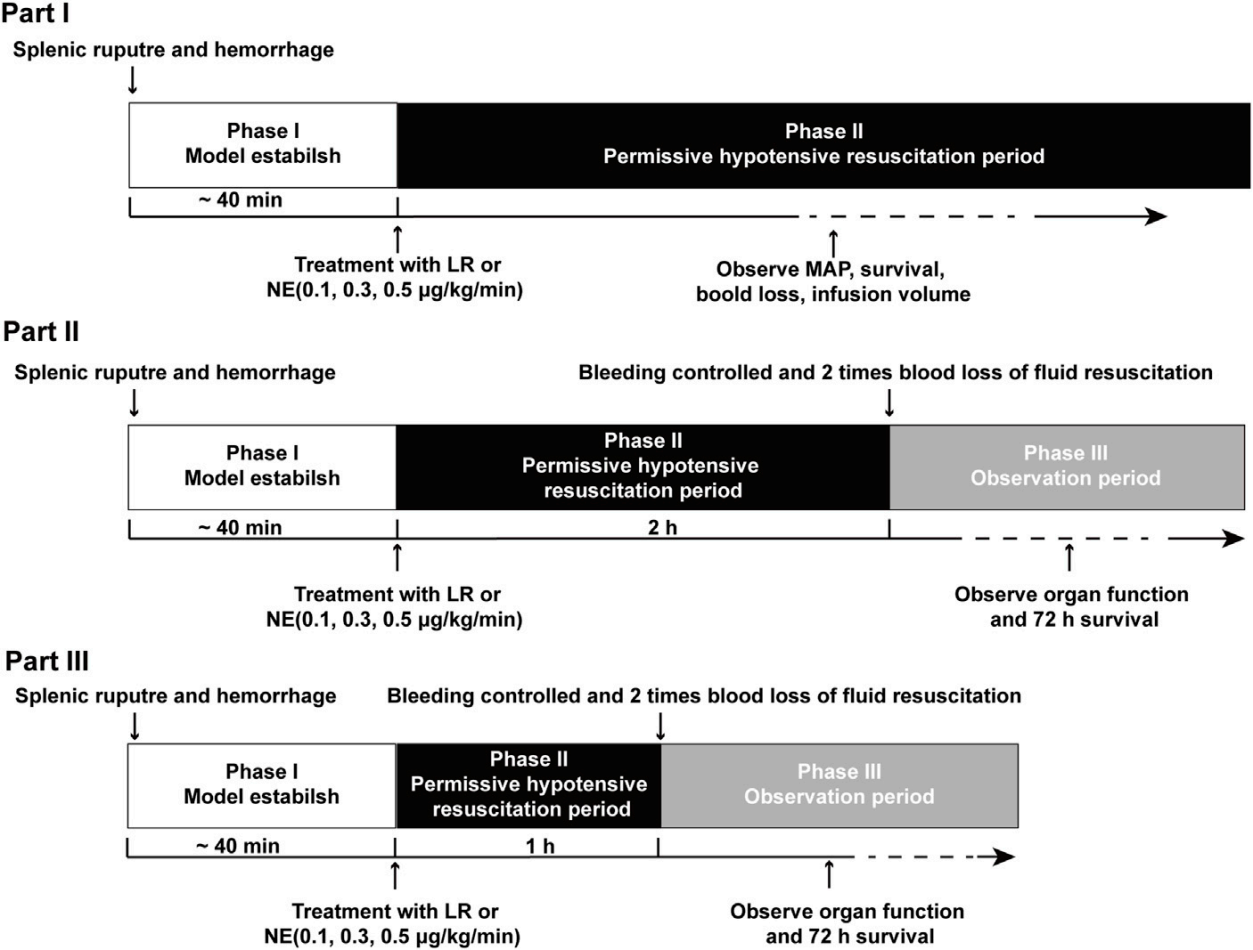
Timeline of the experimental phases. Phase I was the UHS period. Phase II was the permissive hypotensive resuscitation period. Phase III was the observation period. Part I: effects of NE administration on tolerance time during the permissive hypotensive resuscitation period before bleeding was controlled following UHS. Part II: the effects of NE on organ function and survival when the splenic artery was ligated after NE administration for 2 h during the hypotensive resuscitation period. Part III: the beneficial effects of NE on hemodynamics, organ function, and survival when the splenic artery was ligated after NE administration for 1 h.

All experiments were conducted in three parts (Figure 1). The first part aimed to investigate the effects of NE during permissive hypotensive resuscitation on the golden treatment time for UHS. According to the results of the first part, the second part aimed to investigate the effects of NE on organ function and survival time following hypotensive resuscitation for 2 h. The third part aimed to further investigate the beneficial effects of NE on subsequently definitive treatment following hypotensive resuscitation for 1 h. The hemodynamics, cardiac function, and tissue blood flow were observed.

Part I: To investigate if NE can buy golden treatment time in UHS, the experiments were divided into two phases. Phase I was the model stage (UHS), in which blood flowed freely into the abdominal cavity. When the MAP decreased to 40 mmHg, the UHS model was established. Phase II was the permissive hypotensive resuscitation period. In phase II, rats received permissive hypotensive resuscitation with LR, 0.1, 0.3, or 0.5 μg/kg/min NE at a designed MAP of 50–60 mmHg until the rats died. The transected spleen was not ligated during the entire process (Figure 1). Blood loss, infusion volume, MAP, and survival time were recorded.

Part II: To investigate the effects of NE on prolonging the golden time of early treatment for UHS, the experiments were divided into three phases. Phase I was also the model stage (UHS). Phase II was the permissive hypotensive resuscitation period. According to the results of part I, rats in the LR group could not maintain the 50–60 mmHg MAP over 2 h, and most rats in the NE group could maintain target MAP over 2 h, so 2 h was selected as hypotensive maintenance time in this part. In phase II, rats received permissive hypotensive resuscitation with 0.1, 0.3, or 0.5 μg/kg/min NE at a designed MAP of 50–60 mmHg for 2 h. Phase III was the definitive treatment period. Bleeding was stopped after 2 h of hypotensive resuscitation by full ligation of the splenic artery, then rats were resuscitated with 2 times the volume of blood loss with LR. Then the liver function, kidney function, cardiac function, and survival time were measured after a 2-h observation period.

Part III: To further investigate the beneficial effects of NE on UHS, the experiments were also divided into three phases. The groups and fluid infusion procedures were the same as those in part II, while the splenic artery was ligated at 1 h of hypotensive resuscitation. In addition, the hemodynamics, cardiac function, and blood flow of liver/kidneys and their function, animal survival time, and numbers were observed.

### Parameters measurement

#### Animal survival, blood loss, and fluid requirement

Sprague-Dawley rats were randomly divided into 5 groups (*n* = 16 per group): UHS, LR, 0.1 μg/kg/min NE (0.1 NE), 0.3 μg/kg/min NE (0.3 NE), and 0.5 μg/kg/min NE (0.5 NE). At the end of Phase II, blood loss was recorded. The volume of fluid infusion during Phases II and III were also noted. At the end of Phase III, the catheters were removed, the incisions were closed, and the animal survival time was observed up to 72 h after the onset of Phase III.

#### Blood gas, oxygen delivery, and oxygen use

Blood gas, MAP, heart rate (HR), and cardiac output (CO) were measured at baseline, at the end of UHS, the permissive hypotensive resuscitation, and the definitive resuscitation period as described previously (Zhu et al., 2019). Blood gases with 0.2 ml blood were determined using a blood gas analyzer (Phox plus L; Nova Biomedical, Waltham, MA). MAP and HR were measured through the right femoral artery catheter with a polygraph physiological recorder (SP844; Power Laboratory, AD Instruments). CO was measured by thermodilution with the cardiac blood transfusion apparatus (Cardio MAX, model 0162-004M). Cardiac index (CI), stroke index (SI), tissue oxygen supply (DO_2_), and oxygen consumption (VO_2_) were calculated by following formulas: CI=CO/S (S was body surface area), $S=K\times W^{2/3}$ (K = 9.1, W: weight), SI=CI/HR , $DO_{2}=CI\times 13.4\times Hb\times SaO_{2}$ , $DO_{2}=CI\times 13.4\times Hb\times \left(SaO_{2}-SvO_{2}\right)$ (Zhu et al., 2022b).

#### Blood flow and function of vital organ and mitochondrial function

The blood flow in the liver and kidneys was measured by a Doppler imaging instrument (Peri Cam PSI ZR, Sweden). A three-ml arterial blood sample was withdrawn for the determination of heart, liver, and kidney function (including cardiac function troponin T (TnT), aspartate aminotransferase [AST], alanine aminotransferase [ALT], blood urea nitrogen [BUN], and serum creatinine [Scr]) by a biochemical analyzer (Beckman, Fullerton, CA). Thereafter, the rats were killed by a pentobarbital-based euthanasia solution (Sleepaway, 2 ml, intravenously administered) to remove the heart, liver, and kidneys for the measurement of their mitochondrial function by a mitochondrial function analyzer (MT 200, Strathkelvin, Lanarkshire, Scotland). The mitochondrial function was reflected by the respiration control rate (consumed oxygen rate with and without adenosine diphosphate), which was determined as described in our previous work (Li et al., 2012).

#### Water content of the lung and brain

Rats were killed by injecting an overdose of sodium pentobarbital. The brain and lungs were quickly removed, and stained with blood, weighed (wet weight), dried at 105°C for 72 h, and weighed (dry weight). The calculation formula of water content: (wet weight-dry weight)×100%/wet weight (Zhu et al., 2022a).

### Transmission electronic microscopy observation

The fresh heart was quickly fixed with arsenate buffer containing 2.5% glutaraldehyde (pH = 7.4, 4°C) for 24 h. After three 5-min washes with PBS, the heart was postfixed in 1% OsO_4_ for 2 h at room temperature and then dehydrated in a graded ethanol series (65, 70, 75, 80, and 95% for 10 min each). After that, the heart was incubated with Ter butoxide for 10 min and then dried with CO_2_, stained with uranyl acetate, and coated with gold (Au) using an ion sputter coater. Finally, samples were viewed and imaged with a transmission electron microscope (TEM) (H-7700, Hitachi Company, Japan).

### Statistical analyses

Data are the mean ± SD of n observations. The statistical differences among the groups were analyzed by one-way or two-way ANOVA, followed by the post-hoc Tukey test for multiple comparisons between two groups. The animal survival time was analyzed by median and interquartile ranges. Data processing was performed with SPSS 20.0 statistical analysis software package. A *p* value less than 0.05 was considered significant.

## Results

### NE prolonged the golden treatment time for uncontrolled hemorrhagic shock

#### MAP

**FIGURE 2:**
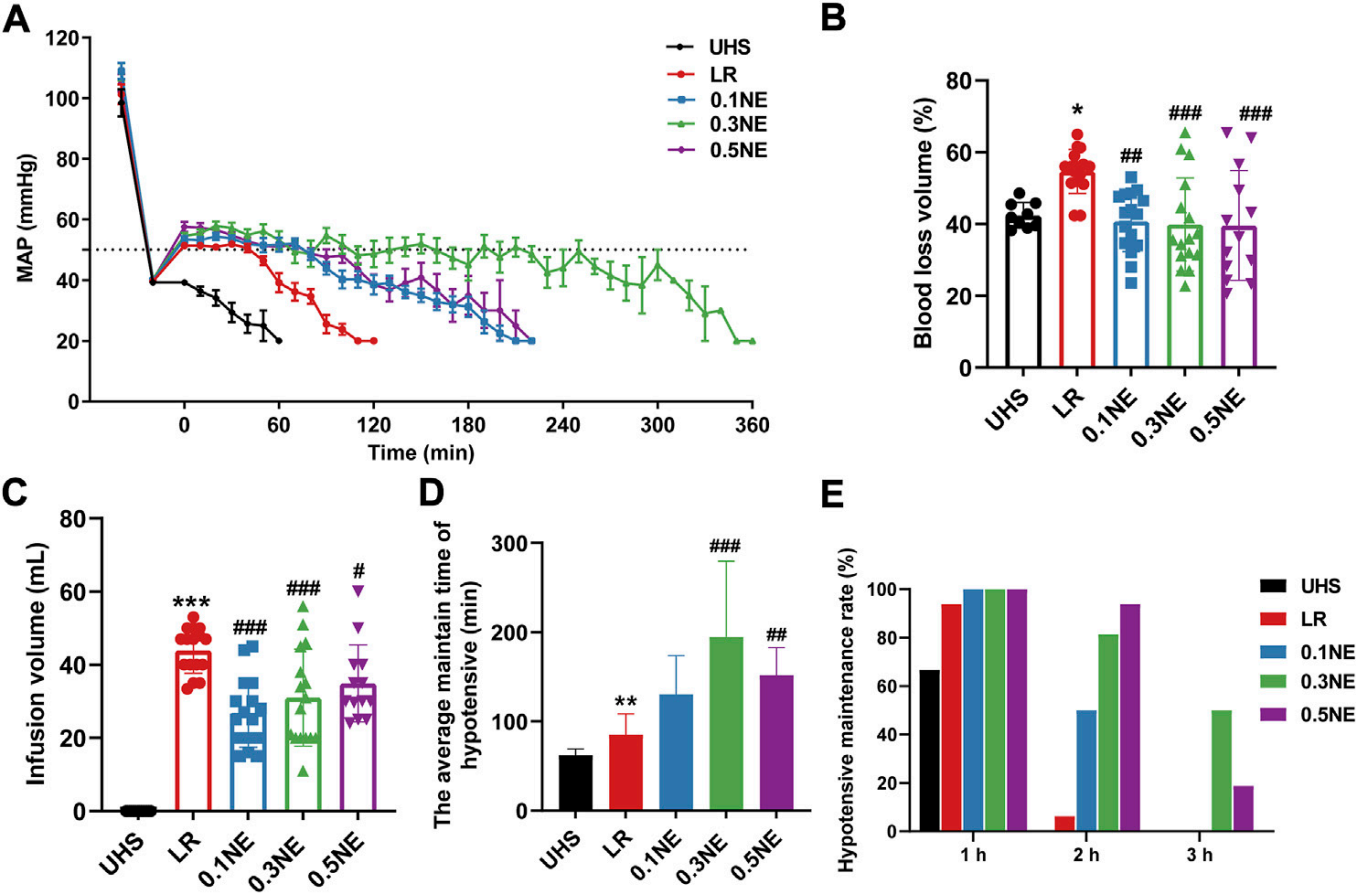
NE prolonged the golden treatment time for UHS. **(A)** Mean arterial pressure (MAP). **(B,C)** Blood loss and infusion volume in permissive hypotensive maintenance period (Phase II). **(D)** The hypotensive maintenance time of NE or LR. **(E)** The hypotensive maintenance rate of rats with UHS at different times. ^*^ *p* < 0.05, ^**^ *p* < 0.01, ^***^ *p* < 0.001 as compared with UHS. ^#^ *p* < 0.05, ^##^ *p* < 0.01, ^###^ *p* < 0.001 as compared with LR.

To explore the effect of NE on the golden treatment time for UHS, the rats received permissive hypotensive resuscitation with LR, 0.1, 0.3, or 0.5 μg/kg/min NE at a designed MAP of 50–60 mmHg, and the MAP, blood loss, infusion volume, and hypotensive maintenance time were recorded. The results showed that the MAP of rats was decreased to 40 mmHg within 30–40 min after the spleens were cut off. LR alone infusion could effectively maintain MAP to 50–60 mmHg for 1 h; the MAP decreased gradually after 1 h, and MAP could not maintain the designed level even with increasing infusion volume. The MAP of most rats decreased to a very low level (<20 mmHg) at 90 min. NE administration during hypotensive resuscitation could maintain MAP at designed 50–60 mmHg over 2 h. NE (0.3 μg/kg/min) had the best maintenance effect, with 37.5% (6/16) of rats maintaining MAP at 50–60 mmHg for 3 h (Figure 2A).

#### Blood loss and infusion volume

In order to prevent the influence of blood coagulation on blood loss calculation, blood loss was calculated when the MAP was less than 20 mmHg, and the infusion volume was recorded at the same time. With LR resuscitation alone, the rate of blood loss was 54.7% ± 6.12%. Different doses NE (0.1, 0.3, 0.5 μg/kg/min) significantly reduced blood loss during the hypotensive maintenance period in rats with UHS as compared with the LR group; they were 40.76% ± 8.46%, 39.87% ± 12.98%, and 39.58% ± 15.29%, respectively (Figure 2B). Correspondingly, the infusion volume also showed a similar trend with blood loss (Figure 2C).

#### Hypotensive resuscitation maintenance time

The results showed that when the MAP dropped to 20 mmHg, increasing the infusion of NE or LR could not improve the blood pressure. Therefore, it was no longer resuscitated when MAP≤20 mmHg, and the time was regarded as the hypotensive resuscitation maintenance time. The average hypotensive resuscitation maintenance time was 85.31 ± 22.98 min in the LR group. NE (0.1, 0.3, 0.5 μg/kg/min) administration prolonged the hypotensive maintenance time significantly; they were 130.3 ± 43.53 min, 194.7 ± 85.04 min, and 151.9 ± 30.98 min, respectively (Figure 2D). 93.73% (15/16) of the rats could maintain MAP at 50–60 mmHg for 1 h in the LR group, while only 37.5% (6/16) of the rats could maintain more than 1.5 h, and only one rat maintained 2 h. NE administration could significantly improve the maintenance rate for MAP at 50–60 mmHg level for 2 and 3 h. In the NE (0.1 μg/kg/min) group, 50% (8/16) of the rats could maintain MAP at 50–60 mmHg for more than 2 h. In the NE (0.3 μg/kg/min) and NE (0.5 μg/kg/min) groups, 50% and 18.75% of the rats maintained MAP at 50–60 mmHg for 3 h, respectively (Figure 2E).

### Effects of NE on organ function and survival time following prolonging the golden treatment time for 2 h

#### Blood loss and infusion volume

**FIGURE 3:**
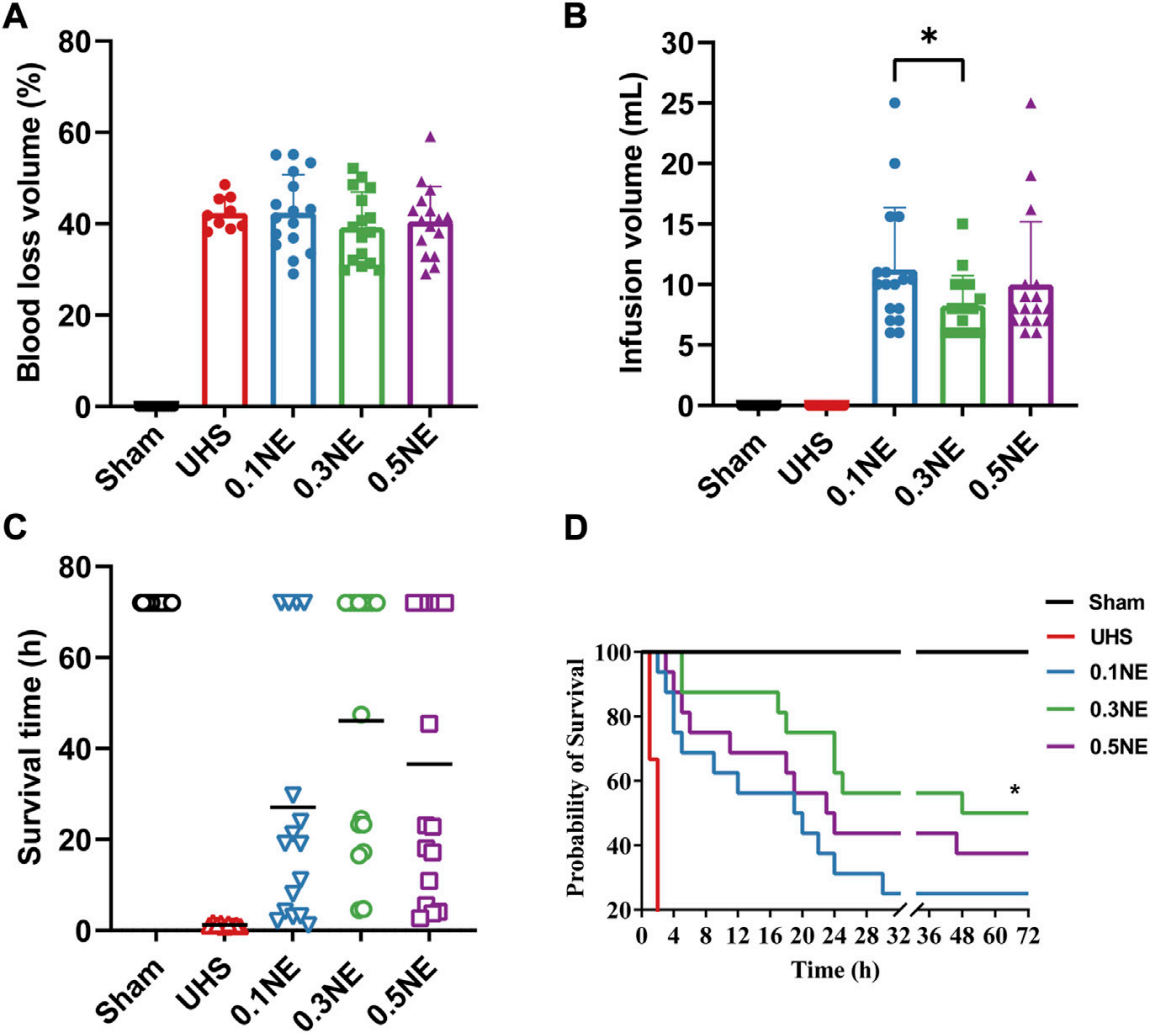
The effect of NE on survival time following prolonged golden treatment time for 2 h. **(A,B)** Blood loss and infusion volume in the permissive hypotensive maintenance period (Phase II). ^**^, *p* < 0.01. **(C)** The average survival time. **(D)** 72-h survival rate. ^*^, *p* < 0.05 as compared with UHS.

According to the above results, NE administration during hypotensive resuscitation can prolong the prehospital treatment “golden time” of UHS to 2–3 h. Then, the effects on organ function and survival were observed when NE was applied to prolong hypotension resuscitation time. Following hypotensive resuscitation for 2 h, the spleen was ligated to stop bleeding, and the effects on vital organ functions and survival were observed. The LR group was not set in this part because the hypotension resuscitation time infusing LR alone was less than 2 h. The results showed that 0.3 μg/kg/min NE administration could maintain MAP at 50–60 mmHg for 2 h before the bleeding was controlled, and the rate of blood loss was 39.22% ± 7.72%, which was significantly lower than the 0.1 μg/kg/min NE group (42.46% ± 8.33%). The rate of blood loss in the NE (0.5 μg/kg/min) group was 40.54% ± 7.65% (Figure 3A). Correspondingly, fluid requirements in the NE (0.1 μg/kg/min) and NE (0.5 μg/kg/min) groups were more, and the fluid requirements were 11.24 ± 5.13 ml and 10.01 ± 5.18 ml, respectively, but the fluid requirement of the NE (0.3 μg/kg/min) group was only 8.25 ± 2.48 ml (Figure 3B).

#### Survival

The shock rats died within 2 h if they were not resuscitated. After 2-h hypotensive resuscitation with NE, the average survival times were significantly prolonged, which were 27.10 ± 28.10 h, 46.09 ± 28.32 h, and 36.60 ± 30.11 h in the 0.1, 0.3, and 0.5 μg/kg/min NE groups, respectively (Figure 3C). The 72-h survival rate in the NE (0.3 μg/kg/min) group was 50% (8/16), significantly higher than that in the NE (0.1 μg/kg/min) group (25%, 4/16). In the NE (0.5 μg/kg/min) group, 37.5% (6/16) of the rats survived for more than 72 h (Figure 3D).

#### Organ function

**FIGURE 4:**
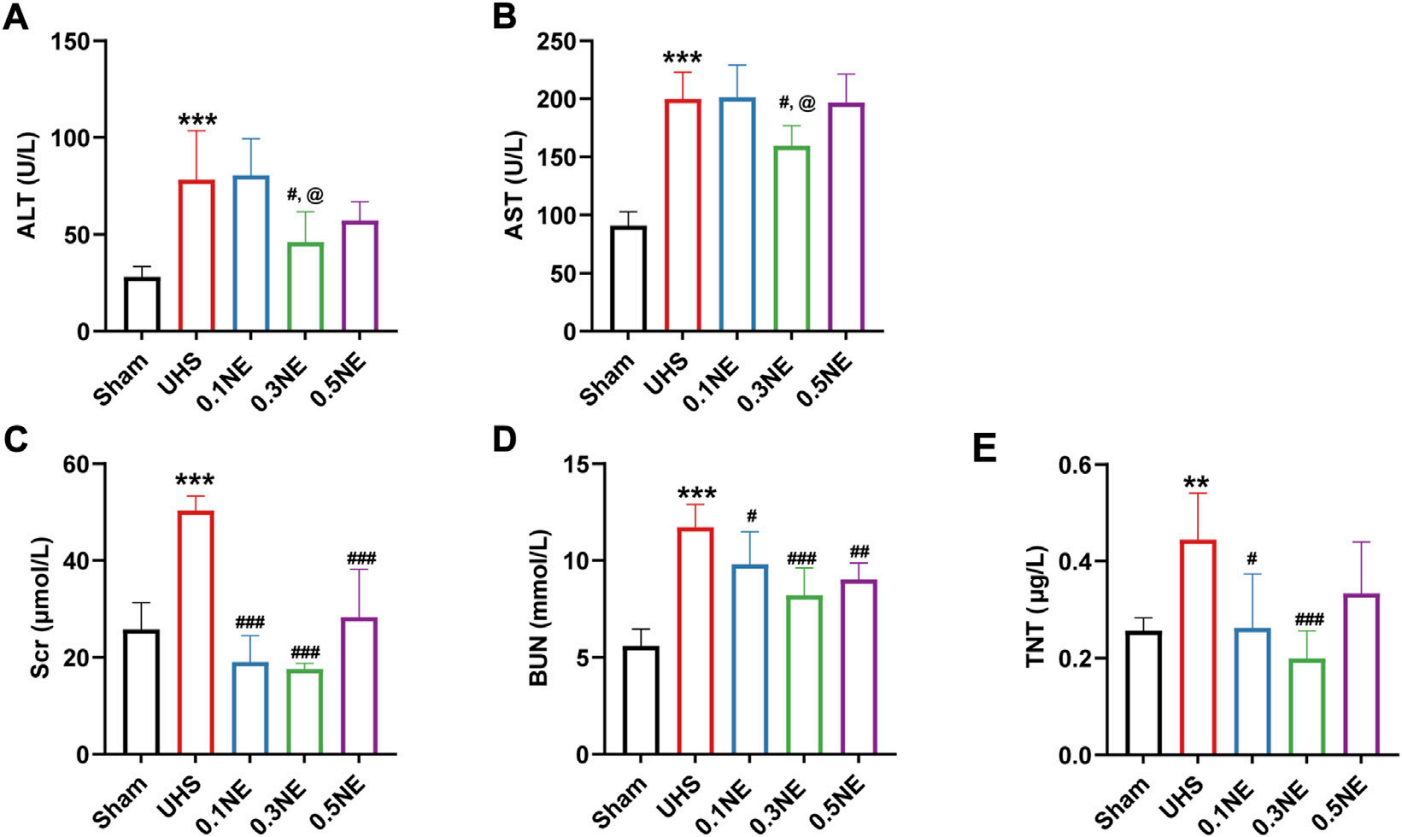
The effect of NE on vital organ functions following prolonged golden treatment time for 2 h. **(A)** ALT. **(B)** AST. **(C)** Scr. **(D)** BUN. **(E)** TnT. ^**^ *p* 0.01, ^***^ *p* < 0.001 as compared with the sham group; ^#^ *p* < 0.05, ^##^ *p* < 0.01, ^###^ *p* < 0.001 as compared with the UHS group; ^@^ *p* < 0.05 as compared with the 0.1 NE group.

The TnT, AST, ALT, Scr, and BUN were significantly increased following UHS in rats, indicating that the heart, liver, and kidney functions were severely damaged. The damage of the heart, liver, and kidney functions was not aggravated after 2-h hypotensive resuscitation with different dose NE, and followed by hemostasis and definitive treatment. On the contrary, compared with the UHS group, the damage of heart, liver, and kidney functions were alleviated in the NE (0.3 μg/kg/min) group (Figure 4). These results indicated that NE administration during the hypotensive resuscitation period can significantly prolong the golden treatment time for prehospital, protect vital organ functions, and improve survival.

### Protective effect of norepinephrine on uncontrolled hemorrhagic shock following 1 h of hypotensive resuscitation

The above results showed that both LR alone and NE plus LR could maintain the MAP at 50–60 mmHg for 1 h after UHS, when the effects of NE and LR on UHS were further compared. Following 1-h hypotensive resuscitation with LR or NE, hemostasis and definitive treatment were followed, and then the effects for UHS were observed.

#### Blood loss and infusion volume

**FIGURE 5:**
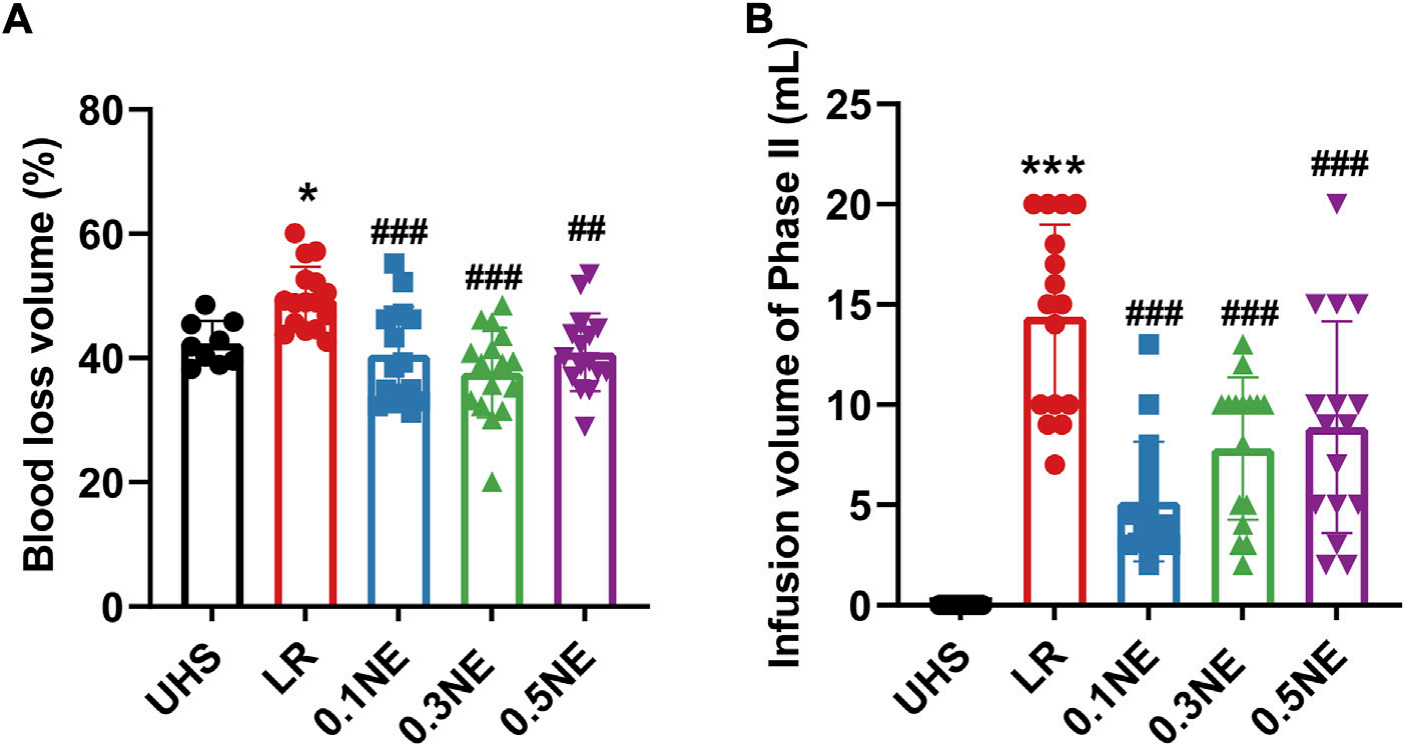
The effect of NE on blood loss and infusion volume following 1 h of hypotensive resuscitation. **(A,B)** Blood loss and infusion volume in the permissive hypotensive maintenance period (Phase II). ^*^ *p* < 0.05, ^***^ *p* < 0.001 as compared with the UHS group; ^##^ *p* < 0.01, ^###^ *p* < 0.001 as compared with the LR group.

With 1-h hypotensive resuscitation with LR alone, the rate of blood loss was 49.58% ± 5.1%, NE administration decreased blood loss for UHS, and the rates of blood loss were 40.53% ± 7.69%, 37.64% ± 7.21%, and 40.91% ± 6.23% in the 0.1, 0.3, and 0.5 μg/kg/min NE groups, respectively. At the same time, the infusion volume of each NE group was decreased, only about half of the LR group (Figure 5).

#### Oxygen delivery and consumption

**FIGURE 6:**
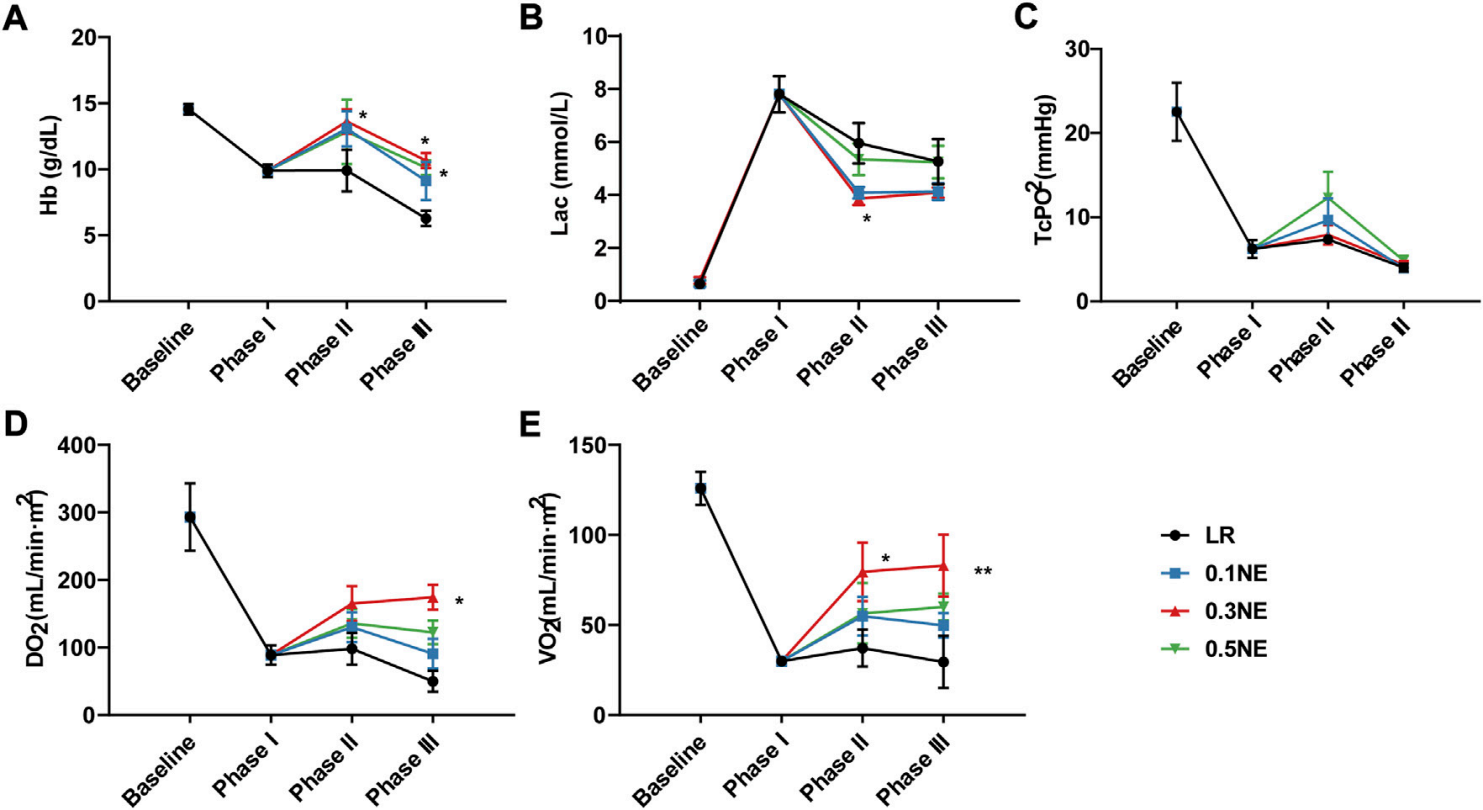
The effect of NE on oxygen delivery and consumption following 1 h of hypotensive resuscitation. **(A)** Hb. **(B)** blood lactate. **(C)** TcPO_2_. **(D)** DO_2_. **(E)** VO_2_. ^*^ *p* < 0.05, ^**^ *p* < 0.01 as compared with the LR group.

NE is well known as a vasoconstrictor. Whether NE inducing the contraction of the peripheral vascular disturbs the oxygen delivery and consumption is not clear. Following UHS, Hb content, and transcutaneous oxygen partial pressure, DO_2_ and VO_2_ were significantly decreased, and the blood lactate level was significantly increased. LR treatment alone did not ameliorate on the above parameter during the whole process, but Hb content further decreased at the end of phase III (observation period). Different doses of NE administration did not disturb the tissue oxygen delivery and consumption. On the contrary, the Hb content in the NE group was increased due to the reduction of blood loss and blood lactate, and DO_2_ and VO_2_ also improved significantly, especially in the NE (0.3 μg/kg/min) group (Figure 6).

#### Function of heart, liver, and kidneys

**FIGURE 7:**
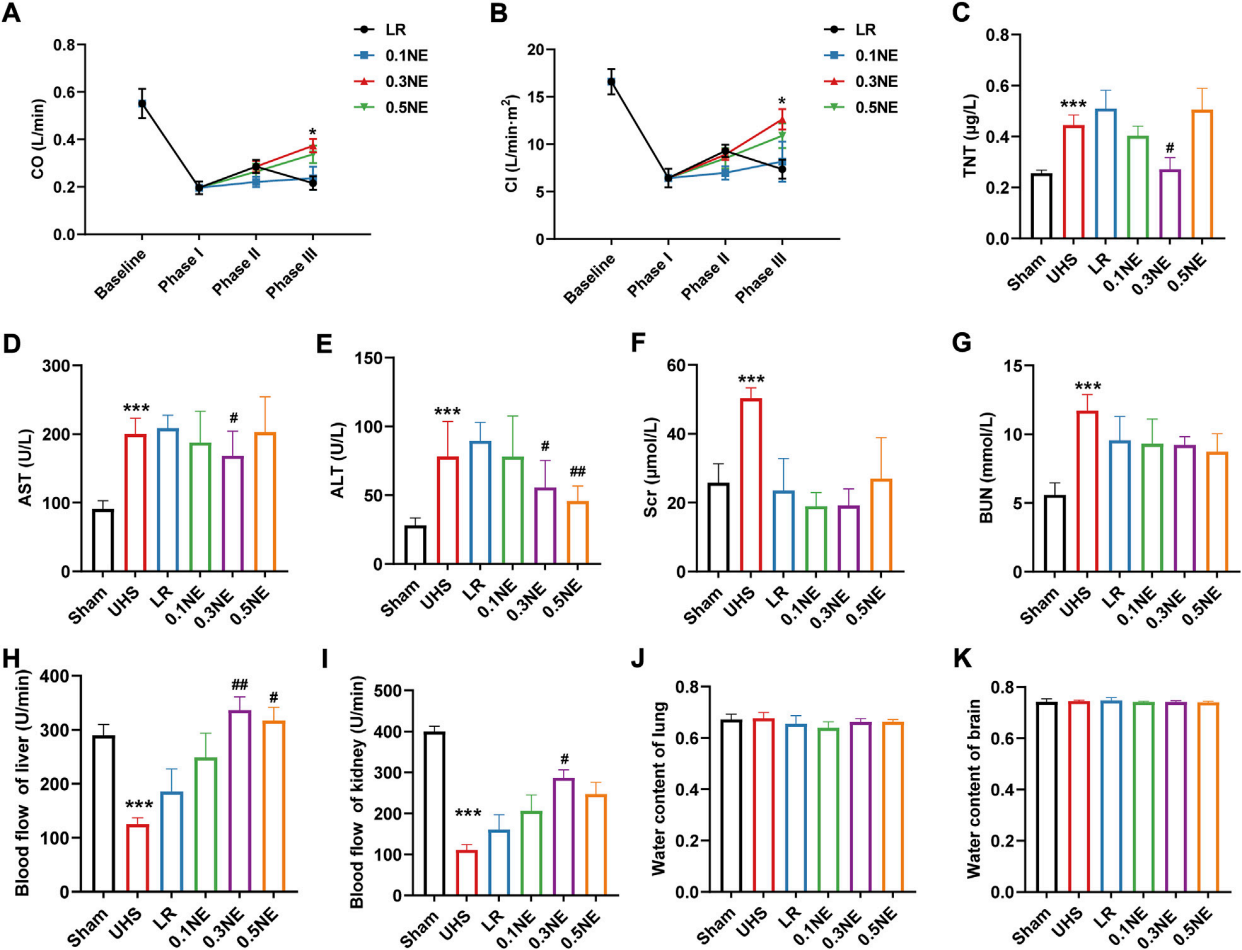
The effect of NE on vital organ functions following 1 h of hypotensive resuscitation. **(A)** CO. ^*^ *p* < 0.05 as compared with LR group. **(B)** CI. ^*^ *p* < 0.05 as compared with LR group. **(C)** TnT. ^***^ *p* < 0.001 as compared with the Sham group. ^#^ *p* < 0.05 as compared with the LR group. **(D,E)** AST and ALT. ^***^ *p* < 0.001 as compared with the Sham group. ^#^ *p* < 0.05, ^##^ *p* < 0.01 as compared with LR group. **(F,G)** Scr and BUN. ^***^ *p* < 0.001 as compared with the Sham group. **(H,I)** Blood flow of liver and kidney. ^***^ *p* < 0.001 as compared with the Sham group. ^#^ *p* < 0.05, ^##^ *p* < 0.01 as compared with LR group. **(J,K)** water content of the lung and brain.

CO, CI, and TnT were used to evaluate cardiac function. Similarly, at the end of the shock, the cardiac function was severely impaired, as manifested by the decrease of CO and CI and the increase of TnT. Resuscitation with LR alone could not improve the cardiac function. Following NE treatment, CO and CI were recovered to a certain extent, and NE (0.3 μg/kg/min) could restore it to 67% and 75% of normal level, significantly higher than those of the LR group (Figures 7A,B). Compared with the LR group, TnT was significantly decreased in the NE (0.3 μg/kg/min) group, indicating that NE (0.3 μg/kg/min) could alleviate cardiac injury in rats (Figure 7C).

Furthermore, AST, ALT, Scr, and BUN were significantly increased at the end of the shock, and the LR only infusion had no beneficial effect on the liver damage induced by UHS (Figures 7D–G). However, the liver damage in rats was alleviated in the NE (0.3 μg/kg/min) group, as well as the kidneys.

On the other hand, blood flow in the liver and kidneys was significantly reduced at the end of the shock, at only 43% and 27% of the baseline level, respectively (Figures 7H,I). Hypotensive resuscitation with LR alone increased blood flow both in the liver and kidneys to some extent (64% and 40%, respectively). The effects of NE (0.1 μg/kg/min) on liver and kidney blood flow were similar to those in the LR group. Hypotensive resuscitation with NE (0.3 μg/kg/min) or NE (0.5 μg/kg/min) significantly improved the hepatic blood flow to the baseline level. NE (0.3 μg/kg/min) also improved the renal blood flow to 72% of the baseline level. UHS did not induce the disorder of the water content of the lung and brain, and neither LR alone nor with NE did not disturb the water content of lung and brain (Figures 7J,K).

#### Mitochondrial function of vital organs

**FIGURE 8:**
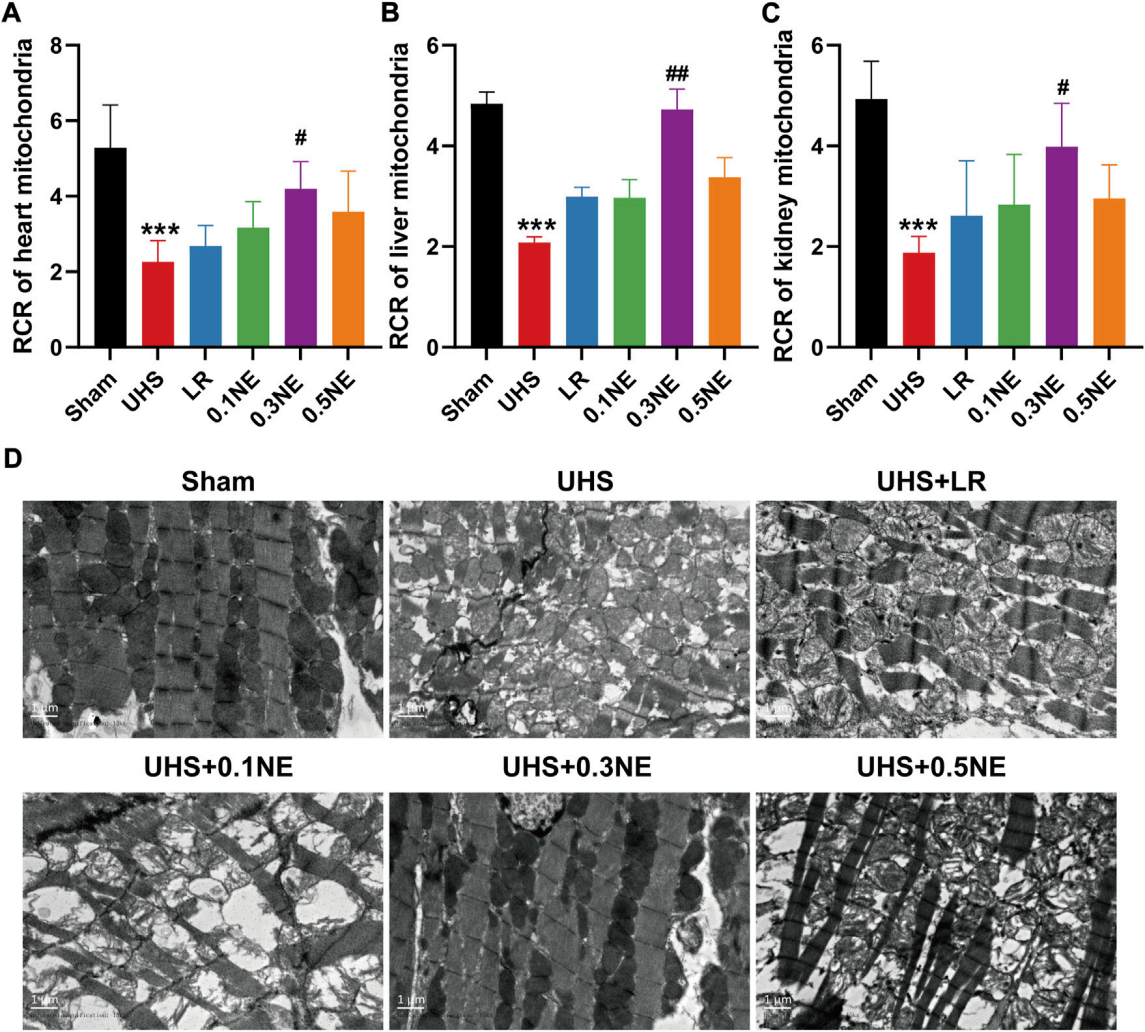
The effect of NE on mitochondrial function of the vital organ following 1 h of hypotensive resuscitation. **(A–C)** The RCRs of mitochondria in heart, liver, and kidneys. ^***^ *p* < 0.001 as compared with the Sham group. ^#^ *p* < 0.05, ^##^ *p* < 0.01 as compared with the LR group. **(D)** Mitochondrial morphology of heart by transmission electron microscope (TEM).

Mitochondrial function determines organ function. The effect of LR or NE on mitochondrial function in vital organs including heart, liver, and kidneys were further observed. At the end of the shock, RCRs of mitochondria in the heart, liver, and kidneys were significantly reduced, to only 50% of the baseline level. Hypotensive resuscitation with LR alone could not improve the mitochondrial function. After hypotensive resuscitation with NE (0.3 μg/kg/min), the RCRs of mitochondria in the heart, liver, and kidneys were significantly increased, up to 77%, 97%, and 79%, respectively (Figures 8A–C). Mitochondrial structure plays a crucial role in mitochondrial function, and the effects on mitochondrial morphology were further observed in the present study. Heart mitochondria were orderly distributed along the sarcomere in the sham group. Following UHS, mitochondria were disordered and swelling, and the sarcomere and crista structure disappeared. The mitochondrial morphology was partly improved following the LR treatment alone. After NE (0.3 μg/kg/min) administration, the mitochondrial morphology and structure in the cardiac muscle were notably restored with evenly arrangement and integral morphology (Figure 8D).

#### Survival

**FIGURE 9:**
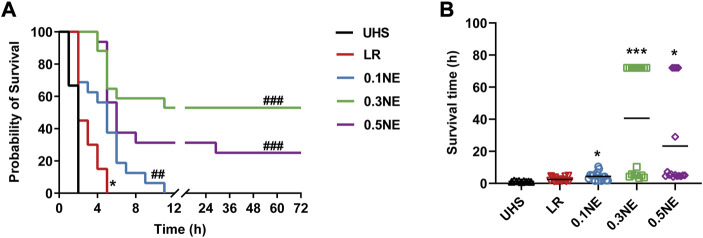
The effect of on survival outcome following 1 h of hypotensive resuscitation. **(A)** 72 h survival rate. ^*^ *p* < 0.05 as compared with the UHS group. ^##^ *p* < 0.01, ^###^ *p* < 0.001 as compared with the LR group. **(B)** the avrage survival time. ^*^ *p* < 0.05, ^***^ *p* < 0.001 as compared with the LR group.

In LR group, the average survival time was 2.45 ± 1.23 h, and the longest survival time was 5 h. One-hour hypotensive resuscitation with three doses of NE could prolong the survival time (4.37 ± 2.64 h in 0.1NE group, 40.59 ± 34.37 h in 0.3NE group, and 23.28 ± 29.66 h in 0.5NE group). The longest survival time in the NE (0.1 μg/kg/min) group was 10.4 h, while there were 9 (52.94%) and 4 (25%) of 16 rats in the NE (0.3 μg/kg/min) and NE (0.5 μg/kg/min) groups that survived for more than 72 h, respectively (Figure 9).

## Discussion

Early treatment of hemorrhagic shock is of great significance to improve the survival rate of trauma. Early and rapid fluid resuscitation can restore the efficient circulating blood volume to increase vital organ perfusion. In recent years, studies showed that permissive hypotensive resuscitation during early treatment for active bleeding was a profitable measure, which could reduce the bleeding and maintain the basic needs of the body until the hemorrhage is controlled. However, it was about 1 h of tolerance time for hypotensive resuscitation, and this could not meet the needs of evacuation in remote mountainous areas. There is an urgent need to find strategies to prolong the golden treatment time. Vasoactive drugs are often used to elevate blood pressure, such as NE, but most of these drugs were recommended in the precondition of adequate volume fluid infusion in a clinic (Wieruszewski and Khanna, 2022). Whether they can be suitable for permissive hypotensive resuscitation at the “prehospital stage” has not been reported. Our present study showed that low-dose NE during the hypotensive resuscitation period could prolong the golden time to 2–3 h. Low-dose NE administration could significantly reduce total blood loss and fluid requirements, improve hemodynamics, and restore oxygen delivery and consumption, as well as alleviate the damage of vital organs including heart, liver, and kidneys, and decrease the early death of animals with UHS. This study provided a proper strategy for the early treatment of trauma.

Our previous studies and other teams demonstrated that permissive hypotensive resuscitation was an effective strategy for hemorrhagic shock before bleeding was controlled, but 90 min during hypotensive resuscitation was the maximal tolerance time for UHS. The evacuation time was often more than 90 min in remote regions or disaster situations. Our previous study found that δ opioid receptor antagonist ICI 174,864 alone or with a small volume of fluid infusion had a beneficial effect on UHS. The ICI 174,864 could effectively prolong the hypotensive resuscitation time to 2 h, improve hemodynamic, oxygen delivery, and tissue perfusion of hemorrhagic-shock rats (Liu et al., 2013). Another study revealed that AVP plus NE could maintain MAP at 55 mmHg for 3 h and “win time” to bring about definitive treatment for UHS. In this study, we found that low-dose NE during the hypotensive resuscitation period could prolong the tolerance time of hypotensive resuscitation. Following NE administration, the hypotensive tolerance time could be extended to 180 min in most animals, and the longest time was up to 300 min for maintaining the MAP at 50 mmHg. These results suggested that low-dose NE provided a longer safe time to reach trauma patients. This could be a proper strategy for the early treatment of trauma patients with active bleeding, especially in remote areas, or military or disaster situations. Furthermore, compared with δ opioid receptor antagonist or AVP + NE, NE has been widely used in clinic as a safe and effective anti-shock drug. This further provided a basis for the clinical application of NE during hypotensive resuscitation at the “prehospital stage.”

Our present study showed that NE administration had a significant improvement in tissue oxygen delivery and consumption, as well as vital organ functions. A similar result was observed in some other studies. Jakobsen et al. (2022) found that early treatment with NE (0.03 mg/kg for 90 min) during severe and hemorrhagic shock restored MAP and chemical variables related to cerebral energy metabolism, and treatment with NE in hypovolemic shock did not cause cerebral vasoconstriction and hypoperfusion. In hemorrhagic shock pigs, Libert et al. (2022) found that resuscitation with a combination of NE at two doses (moderate dose: mean rate of 0.64 µg/kg/min and high dose: mean rate of 1.57 µg/kg/min) and fluid restored renal microcirculation and oxygenation, as well as renal function, in a manner comparable to fluid resuscitation alone and without differences between the two NE doses. However, the mechanism of low-dose NE increasing tissue oxygen delivery and consumption was uncertain.

Due to its pharmacological properties, NE administration has several hemodynamic effects: it increases venous return due to its venoconstrictive effect; it increases cardiac inotropism due to its β-agonist effect; and it increases arterial vasomotor tone by α-agonist effect. High-dose NE has a risk of excessive vasoconstriction, which may cause worsening of coagulation directly or by decreasing tissue perfusion (Cardinale et al., 2020). In the present study, NE (0.1, 0.3, 0.5 μg/kg/min) administration could decrease blood loss while limited fluid needs to achieve a MAP level of 50–60 mmHg. Compared with the LR group, blood loss in the NE group was decreased, suggesting that low-dose NE could reduce the hemorrhage of the splenic artery through its lightly arterial vasoconstriction effect to maintain hemodynamics. Further study showed that low-dose NE led to a decrease of tissue oxygen delivery and consumption. The DO_2_ and VO_2_ were significantly increased in rats after NE treatment, due to decreased blood loss and increased venous return. Moreover, following NE treatment, CO and CI were significantly restored due to the β-agonist effect of NE, then subsequently restoration of volume with fluid resuscitation not only restored splanchnic perfusion but also corrected oxygen debt during experimental hemorrhage. Among these doses, NE (0.3 μg/kg/min) had the best effect in improving the hemodynamics and survival outcome for hemorrhagic shock rats. This may be due to a stronger vasoconstrictive effect with 0.3 μg/kg/min NE than 0.1 μg/kg/min NE, then hemodynamics was maintained by controlling bleeding rapidly. However, 0.5 μg/kg/min NE had the strongest vasoconstrictor effect with a risk of excessing vasoconstriction, which may cause tissue hypoperfusion. It was supported by Cardinale’s (2020) study which showed a maximum NE infusion rate >0.6 μg/kg/min associated with a higher SOFA score at 24 h (Cardinale et al., 2020). Of course, further studies and clinical trials are needed to further confirm the exact dose of NE beneficial for traumatic patients. The present study showed that blood loss led to the decrease of Hb content in blood circulation at the end of the shock (phase I). Following 1-h hypotensive resuscitation with NE (phase II), the Hb content increased as compared with phase I, while it further decreased following LR infusion; the reason was not clear. According to NE’s pharmacological properties, NE could increase arterial vasomotor tone by α-agonist effect, which might squeeze the blood capillary and then induce the blood in the capillaries returning to the blood circulation; this may be the reason that the Hb content increased in phase II in the NE group while not in the LR group. The detailed mechanism needs further study.

Mitochondria are integral to cellular function and are responsible for energy production in cells. Mitochondrial function determines organ function while mitochondrial function is affected by mitochondrial structure. Our previous study found that severe trauma led to an imbalance in mitochondrial mass by increasing ER-mito contact, which in turn caused multiple organ dysfunction. In the present study, UHS induced a significant decrease in the mitochondrial activity of the heart, liver, and kidneys, and a significant increase in the number of cardiac mitochondria. Mitochondrial function and structure were significantly improved after 0.3 μg/kg/min NE treatment, and the damage of heart, liver, and kidneys was lighter than LR treatment alone. These findings suggested that low-dose NE could protect organ function by improving mitochondrial morphology and function during UHS. The most important reason was that the co-administration of NE with fluid during resuscitation not only respected tissue perfusion but also restored oxygen delivery and consumption. Indeed, our previous studies found that high doses of NE could further induce mitochondrial fragmentation by disrupting actin. Some studies also showed that sustained high NE levels led to decreased mitochondrial calcium uptake and increased intracytoplasmic calcium concentration (Gutiérrez et al., 2014), then the communication and proximity between SR/ER and mitochondria were changed (Tappia et al., 2022). That may be the reason why 0.5 μg/kg/min did not significantly attenuate mitochondrial damage induced by UHS.

There are several limitations to our study. First, the animal we used in the present study is a small animal (rat), so whether these results can be extrapolated to large animals or even humans needs further confirmation. Second, although the results showed that NE prolonged the tolerance time during hypotensive resuscitation and increased the safe time taken to reach the patient, the ideal safe time needs further investigation.

In conclusion, the present study showed that low-dose NE was suitable for the early treatment of UHS during hypotensive resuscitation, which could prolong the golden window of emergency treatment time for serious trauma patients by reducing blood loss and protecting the function of vital organs.

## Data Availability

The raw data supporting the conclusion of this article will be made available by the authors, without undue reservation.

## Fundings

This work was supported by the Key Research Projects of Military Logistics of China (Grant No. BLJ19J008).
